# Phenolic Content, Antioxidant Potential, and Antimicrobial Activity of *Uvaria chamae* (Annonaceae), a Food Plant from Burkina Faso

**DOI:** 10.1155/2024/1289859

**Published:** 2024-03-26

**Authors:** Kayaba Kaboré, Crépin Ibingou Dibala, Hemayoro Sama, Mamounata Diao, Marius K. Somda, Mamoudou H. Dicko

**Affiliations:** ^1^Laboratory of Biochemistry, Biotechnology, Food Technology and Nutrition, Department of Biochemistry and Microbiology, University Joseph Ki-ZERBO, Ouagadougou 09 BP 848, Burkina Faso; ^2^Laboratory of Microbiology and Microbial Biotechnology, Department of Biochemistry and Microbiology, University Joseph Ki-ZERBO, Ouagadougou, Burkina Faso

## Abstract

The study aimed to evaluate phenolic content and antioxidant and antibacterial potentials of the fractions of the hydroethanolic extract of *Uvaria chamae* leaves, a food plant from Burkina Faso. Thus, the hexane, dichloromethane, ethyl acetate, and butanol fractions of the hydroalcoholic extract after drying were used to determine phenolic compound content, antioxidant activity, and antimicrobial potential on strains of pathogenic bacteria responsible for food contamination. Phytochemical analyses were performed according to standardized methods, while antioxidant activity was evaluated by DPPH and FRAP methods. The antibacterial activity of the fractions was determined by diffusion and microdilution methods on the agar medium with gentamicin as a reference antibiotic. All the six strains, namely, *Salmonella typhi* ATCC 19430, *Escherichia coli* ATCC 8739, *Staphylococcus aureus* ATCC 25923, *Pseudomonas aeruginosa* ATCC 9027, *Bacillus cereus* ATCC 13061, and *Listeria monocytogenes* ATCC 7644, were sensitive to the fractions tested. Minimum inhibitory concentrations ranged from 37 *µ*g·mL^−1^ to 1.67 mg·mL^−1^, respectively, gentamicin and butanolic fractions, while minimum bactericidal concentrations of the fractions ranged from 0.037 to 2.500 mg·mL^−1^ depending on the bacterial strain. Antioxidant activity varied significantly between fractions. For DPPH free radical scavenging activity, the butanol fraction was the most active, with an IC50 of 280 *μ*g/mL, while the lowest activity (705 *μ*g/mL) was recorded by the hexane fraction. Those of trolox and ascorbic acid used as standards were 80 and 100 *μ*g/mL, respectively. Ferric reducing power (FRAP) ranged from 0.34 to 0.40 mmol EAA/g extract for the hexanic and ethyl acetate fractions, respectively. Phenolic compound contents also varied significantly between fractions. Butanoic and ethyl acetate presented the best contents of total phenolics and flavonoids, respectively. Significant and positive correlations were also recorded between phenolics and antioxidant activities. The antibacterial and antioxidant activities of the active fractions would be related to their richness in bioactive compounds, including phenolic, which are powerful natural antioxidants. *U. chamae* leaf extracts could therefore be used as dietary supplements to boost the immune system and prevent bacterial infections.

## 1. Introduction

Multiple resistance of microorganisms to antimicrobial and particularly to antibiotic drugs in human and plant pathogenic microorganisms has been commonly reported in recent years worldwide, especially in developing countries, due to the indiscriminate use of commercial antibiotics in the treatment of infectious diseases [1]. Also, our lifestyle (smoking, alcoholism, obesity, and intense physical exercise) and our bad eating habits contribute to the production of reactive oxygen species (ROS) in the body called free radicals [2]. The formation of ROS is required for the phenomena of regulation and cellular homeostasis (cellular signaling and apoptosis), but a strong accumulation of these ROS can become toxic because they react with cellular constituents (nucleic acids, proteins, and lipids) particularly when the endogenous antioxidant defenses of the organism (the enzymatic and nonenzymatic defense system) are insufficient. This physiological state called oxidative stress is a cause of the appearance of various pathologies such as diabetes mellitus, cardiac and neurodegenerative diseases, articular pathologies, carcinogenesis, and aging [2].

In light of the evidence of the prevalence of multidrug-resistant isolates, the need to discover new antimicrobial agents assumes undue importance [1]. Many plants have been used because of their antimicrobial activities against pathogenic microorganisms, which are due to phytochemicals synthesized in the secondary metabolism of the plant such as phenolic compounds (flavonoids, tannins, phenolic acids, etc.), saponins, alkaloids, anthraquinones, glycosides, and reducing sugars [3, 4].

To cope with the harmful effects of ROS and the increasing resistance of microbes to antibiotics, it is important to have natural exogenous antioxidant defenses, which should be provided by a healthy diet and/or by medicinal plants. Many plants have been used because of their antioxidant properties and antimicrobial activities against pathogenic microorganisms, which are due to phytochemical compounds synthesized in the secondary metabolism of the plant such as phenolics (flavonoids, saponins, tannins, etc.), alkaloids, anthraquinones, glycosides, and reducing sugars [5]. Various studies conducted on some food and medicinal plants revealed the presence of important constituents, which can be used for pharmacological or pharmaceutical purposes [5]. Researchers extracted many components such as phenols, tannins, flavonoids, alkaloids, and saponins that have antimicrobial, antioxidant, and anti-inflammatory activities. Scientific work is therefore underway to explore the antioxidant and antimicrobial potential of food and medicinal plants [5–7].

*Uvaria chamae* is a food plant of the Burkinabe flora whose fruits are berries with a sweet taste. They are fruits of mouth; i.e., they are consumed raw. *Uvaria chamae* belonging to the Annonaceae family is a large shrub or small climbing tree also known as bush banana or finger root. Its interest for traditional medicine and research of new bioactive molecules lies in the fact that it is widely used by the African population for the treatment of diseases related to oxidative stress [8]. Several studies have reported biological properties such as antitrypanosomal and anti-inflammatory [9], hypoglycemic [10], antidiabetic [11], and antifungal [12] effects against abdominal pain, stomach cramps, side stitch, headache, edema, anemia, and cough, and they are used for wound healing. Also, *Uvaria chamae* in association with other species such as *Dialium guineense*, *Flueggea virosa*, *Cocos nucifera*, and *Morinda lucida* is used as antimalarial and can cure some cancers [13]. Other studies reported the use of *Uvaria chamae* roots against antimicrobial diseases [14–16] and multidrug-resistant strains [17]. Preliminary phytochemical screening reveals the presence of tannins, alkaloids, cardiac glycosides, cyanogenic glycosides, and flavonoids [18, 19]. Also, some authors have linked the antioxidant and antibacterial potential of *U. chamae* to phenolic compounds [19]. Despite this high potential of the species, very little scientific work has been done on the antioxidant and antibacterial properties of the leaves. However, the use of the roots poses enormous ecological problems in the long term. This study aims to contribute to a better knowledge of the antioxidant and antibacterial activities of the leaves of *Uvaria chamae* which could be valued as a dietary supplement in the management of diseases related to toxi-infections but also to the diseases related to oxidative stress.

## 2. Materials and Methods

### 2.1. Biological Materials

#### 2.1.1. Vegetal Material

The vegetal material of the study consisted of fresh leaves of *Uvaria chamae* from the classified forest of Kou (11°10′60″N and 4°27′0″W; latitude: 11.1833°, longitude: −4.4500°), a locality located 15 km northwest of Bobo Dioulasso.

#### 2.1.2. Bacterial Strains

The bacterial strains used in the study were *Bacillus cereus* ATCC 13061, *Listeria monocytogenes* ATCC 7644, and *Staphylococcus aureus* ATCC 25923 for Gram-positive and *Escherichia coli* ATCC 8739, *Pseudomonas aeruginosa* ATCC 9027, and *Salmonella typhi* ATCC 19430 for Gram-negative bacteria.

### 2.2. Sample Collection

After identification of the species in the laboratory of Biology and Plant Ecology of the University Joseph Ki-ZERBO, Burkina Faso, specimens were deposited. Approximately 1 kg of *Uvaria chamae* leaves was collected and transported to the laboratory where they were washed gently with running tap water to remove various contaminants. They were then dried under ventilation. The dried leaves were ground into a fine powder using a mortar. The powder obtained was packed in jars and stored in a cool place at 4°C until use.

### 2.3. Extraction and Fractionation of Extracts

Extraction and fractionation of the extracts were performed following the method described by [20]. Fifty (50) g of *Uvaria chamae* leaf powder was subjected to extraction under magnetic stirring at laboratory room temperature for 48 h with 500 mL of a hydroethanolic mixture (V/V: 20/80). After filtration on filter paper, the filtrate obtained was concentrated using a rotary evaporator at 45°C. The residue of this filtrate was oven-dried for 48 h at 45°C to obtain a dry extract which was used for fractionation. For fractionation, the dry extract of the hydroethanolic macerate was dissolved in 50 mL of distilled water and then subjected to a series of liquid-liquid partitioning with immiscible organic solvents with immiscible organic solvents of increasing polarity such as hexane, dichloromethane, ethyl acetate, and butanol.

### 2.4. Biochemical Analysis

#### 2.4.1. Total Phenolic Content

Total polyphenols were determined by the Folin–Ciocalteu method [21, 22]. Aliquots (125 *μ*L) of solution from extract or each fraction in methanol (10 mg/mL) were mixed with 62.5 *μ*L Folin–Ciocalteu reagent (0.2 N). After 5 min, 500 *μ*L of aqueous Na_2_CO_3_ (75 g/L) was added, and the mixture was vortexed. After 2 h of incubation in the dark at room temperature, the absorbencies were measured at 760 nm against a blank (0.5 mL Folin–Ciocalteu reagent + 1 mL Na_2_CO_3_) on a UV/visible light spectrophotometer (CECIL CE 2041, CECIL Instruments, England). The experiments were carried out in triplicate. A standard calibration curve was plotted using gallic acid (*Y* = 0.029*x* − 0.004; *R*^2^=0.999). The results were expressed as g of gallic acid equivalents (g GAE)/100 g of extract or fractions.

#### 2.4.2. Flavonoid Content

The total flavonoids were estimated according to the Dowd method, slightly modified [22, 23]. An aliquot of 0.5 mL of methanol/AlCl3 (2%, w/v) was mixed with 0.5 mL of extract or each fraction solution (0.1 mg/mL). After 10 min, the absorbencies were measured at 415 nm against a blank (mixture of 0.5 mL extract solutions and 0.5 mL methanol) on a UV/visible spectrophotometer (CECIL CE 2041, CECIL Instruments, England) and compared to a quercetin calibration curve (*Y* = 0.0289*x* − 0.0036; *R*^2^=0.9998). The data obtained were the means of three determinations. The amounts of flavonoids in plant extracts were expressed as g of quercetin equivalents (g QE)/100 g of extract or fractions.

### 2.5. In Vitro Antioxidant Activity

#### 2.5.1. DPPH Radical Method

Radical scavenging activity of extract or each fraction against stable DPPH (2, 2′-diphenyl-1-picrylhydrazyl, Fluka) was determined with a UV/visible spectrophotometer (CECIL CE 2041, CECIL Instruments, England) at 517 nm as described by [22, 23]. Extract solutions were prepared by dissolving 10 mg of dry extract in 10 mL of methanol. The samples were homogenized in an ultrasonic bath. Afterwards, 0.5 mL of aliquots which were prepared at different concentrations from each sample of extract was mixed with 1 mL of methanolic DPPH solution (20 mg/mL). After 15 min of incubation in the dark at room temperature, the decrease in absorption was red. All experiments were performed in triplicate and expressed in mmol of ascorbic acid equivalent per mass of extract or fraction.

#### 2.5.2. Ferric Reducing Antioxidant Power (FRAP) Assay

The FRAP assay was performed as previously described [22, 24]. An aliquot of 0.5 mL of extract or each fraction (1 mg·mL^−1^) was mixed with 1.25 mL of phosphate buffer (0.2 M, pH 6.6) and 1.25 mL of aqueous potassium hexacyanoferrate [K_3_Fe (CN)_6_] solution (1%). After 30 min of incubation at 50°C, 1.25 mL of trichloroacetic acid (10%) was added and the mixture was centrifuged at 2000 × *g* for 10 min. Then, the upper layer solution (0.625 mL) was mixed with distilled water (0.625 mL) and a freshly prepared FeCl3 solution (0.125 mL, 0.1%). Absorbances were read at 700 nm on a UV/visible spectrophotometer (HELIOS EPSILON, THERMO Scientific), and ascorbic acid was used to produce the calibration curve (*Y* = 0.008*x* − 0.0081; *R*^2^=0.9999). The iron (III) reducing activity determination was performed in triplicate and expressed in mmol ascorbic acid equivalent per g of extract or fractions. Ascorbic acid, a reference compound, was used as a positive control.

#### 2.5.3. Antimicrobial Assay of Extracts and Fractions

Antimicrobial assay of extracts and fractions of *U. chamae* was performed by the agar well diffusion method in Mueller–Hinton agar (MHA) plates as described by [4] with minor modifications. The test organisms were inoculated into nutrient broth and incubated overnight at 37°C to adjust turbidity to 0.5 McFarland, resulting in a bacterial suspension with a density of 10^6^-10^7^ CFU/mL. Using sterile forceps, discs consisting of 6 mm diameter Whatman n°1 paper sterilized by autoclaving are placed on the surface of the inoculated agar. A volume of 10 *μ*L of each fraction or extract of 5 and 10 mg/mL concentration in 1% ethanol is deposited on each disc. Gentamicin antibiotic reference discs (120 *μ*g/disc) were used as a positive control, whereas 1% ethanol-impregnated discs were used as a negative control (control: T). The plates were incubated at 37°C for 24 h. Each trial was performed in triplicate. After incubation, the average diameter of the zones of inhibition (mm) produced by the fractions or the positive control (gentamicin) was observed and measured in mm. Strain susceptibility was judged according to the scale of [24]: diameter < 8 mm (not susceptible or resistant), 9 mm ≤ diameter ≤ 14 mm (susceptible), 15 mm ≤ diameter ≤ 19 mm (highly susceptible), and >20 mm (extremely sensitive).

#### 2.5.4. Determination of the Minimum Inhibitory Concentration (MIC) and the Minimum Bactericidal Concentration (MBC)

The broth microdilution method was used to determine the MIC according to CLSI. Twofold serial dilutions of extracts were prepared directly in a microtiter plate containing Mueller–Hinton broth to obtain various concentrations. The positive control was used containing gentamicin as a standard drug. The plate was covered with a sterile sealer and incubated for 24 h at 37°C. The MIC was considered as the lowest concentration of the extract that completely inhibits bacterial growth, while the minimum bactericidal concentration (MBC) corresponds to the concentration where 99.9% of microorganisms have been destroyed.

### 2.6. Statistical Analysis

The analysis of variance (ANOVA) was performed using XL-STAT2016 software to determine the variability of the parameters studied. Tukey's test at the 5% threshold was performed for means comparison.

## 3. Results and Discussion

### 3.1. Results

#### 3.1.1. Fractionation Yields

**Figure 1:**
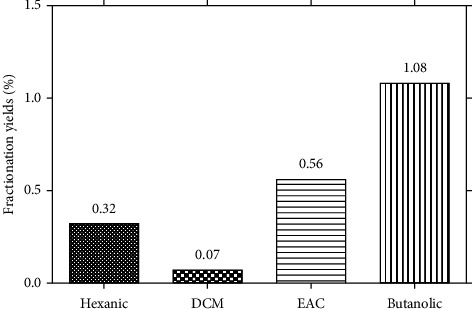
Fractionation yields.

Extraction and fractionation yields (Figure 1) show a variation in yields depending on the fractions. In general, the butanol fraction showed the highest yield (1.08%) followed by the ethyl acetate (EAC) (0.56%) and hexane (0.32%) fractions, while the lowest yields were recorded with the dichloromethane (DCM) fraction (0.07%).

#### 3.1.2. Phenolic Content

Analysis of phenolic composition showed a significant variation in total phenolics and total flavonoids depending on the fractions (Table 1). The contents of total phenolics varied from 15.49 ± 0.18 and 18.33 ± 0.59 mg GAE/100 mg of fraction, while those of flavonoids varied from 1.45 ± 0.12 to 7.26 ± 0.10 mg QE/100 mg. The best content was obtained with the ethyl acetate (18.33 ± 0.59 mg GAE/100 mg fraction) and butanoic (18.13 ± 0.59 mg fraction) fractions, while the hexanic fraction has the lowest content (15.49 ± 0.18 mg GAE/100 mg fraction). For flavonoids, the highest contents were obtained with the butanoic fraction (7.26 ± 0.10 mg QE/100 mg fraction), while the dichloromethane (1.89 ± 0.03 mg QE/100 mg fraction) and hexanic (1.45 ± 0.12 mg QE/100 mg fraction) fractions showed the lowest contents.

#### 3.1.3. Antioxidant Activities of Different Fractions

*(1) DPPH Antiradical Activity*. Linear regression of inhibition percentages as a function of fraction concentrations allowed the determination of IC50s (Table 2). The best IC50 value (280 ± 0.01 *µ*g/mL) of the fractions was obtained with the ethyl acetate (379 ± 0.01 *µ*g/mL) and dichloromethane (657 ± 0.01 *µ*g/mL) fractions, while the hexane fraction showed the lowest activity (705 ± 0.02 *µ*g/mL). Compared with the IC50 of the standards, which are, respectively, trolox and ascorbic acid, these values are relatively high. The fractions show relatively low activity compared with those of the two study standards.

**Figure 2:**
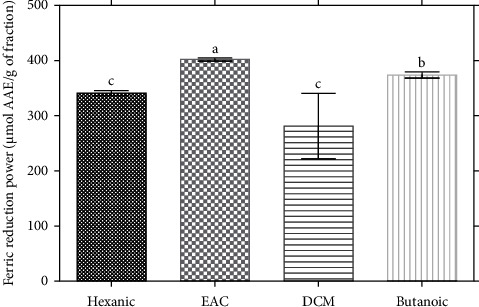
Ferric reduction power. Histograms with different letters (a, b and c) are statistically different at the 5% level.

*(2) Ferric Reduction Power*. The reducing power (Figure 2) of iron shows a significant variation in reducing power depending on the fractions. The reducing power of iron shows a significant variation in reducing power depending on the fractions. The different fractions varied from 340 *µ*mol EAA/g fraction for the hexane fraction to 400 *µ*mol EAA/g for the ethyl acetate (EAC) fraction.

#### 3.1.4. Correlation between Phenolics and Antioxidant Activities

Pearson correlation coefficients recorded showed a positive correlation between total phenolic and flavonoid contents (Table 3). However, positive coefficients were recorded between total phenolic and flavonoid contents (*r* = 0.736) and between phenolic and FRAP activity (*r* = 0.161). Also, negative correlations were recorded between total phenolic content and DPPH IC50 (*r* = −0.736).

#### 3.1.5. Antimicrobial Activity

**Figure 3:**
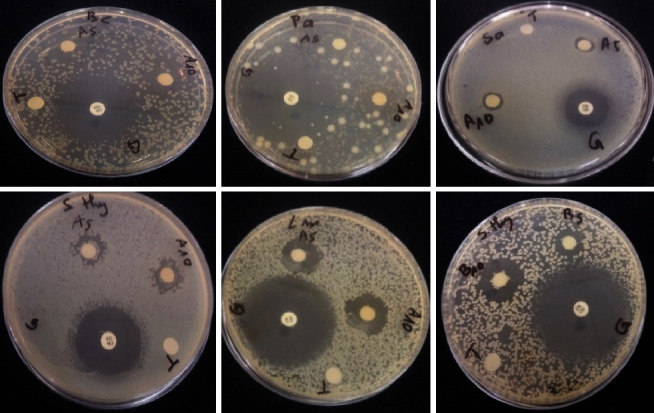
Inhibition diameters of some strains.

*(1) Inhibition Diameters*. The inhibition diameters (Figure 3) of ethyl acetate and butanolic fractions at concentrations of 5 mg·mL^−1^ and 10 mg·mL^−1^ on six bacterial strains were tested in vitro by the agar diffusion method against gentamicin as a reference. Significant variations in inhibition diameters were recorded between certain fractions and the two concentrations tested. Inhibition diameters ranged from 27 to 36 mm for gentamicin. For the ethyl acetate fraction, they ranged, respectively, from 10.5 to 12.33 mm and from 11.16 to 17 mm for concentrations of 5 mg·mL^−1^ and 10 mg·mL^−1^. For the butanol fractions, they ranged from 11.16 to 14 mm and from 12.5 to 18.33 mm for the same concentrations. Interpretation of inhibition diameters (Table 4) shows that all strains are sensitive to both fractions at a concentration of 5 mg·mL^−1^. For the 10 mg·mL^−1^ concentration, *Staphylococcus aureus* ATCC 25923, *Escherichia coli* ATCC 8739, and *Salmonella typhi* ATCC 19430 were sensitive to the ethyl acetate fraction, while the other strains were highly sensitive. For the butanolic fraction at the concentration of 10 mg·mL^−1^, *Bacillus cereus* ATCC 13061, *Listeria monocytogenes* ATCC 7644, and *Salmonella typhi* ATCC 19430 were highly sensitive, while the others were only sensitive (Table 5).

*(2) Minimum Inhibitory and Bactericidal Concentrations*. Minimum inhibitory concentrations for both fractions and gentamicin ranged from 37 *µ*g·mL^−1^ to 1.67 mg·mL^−1^. Gentamicin showed the highest inhibitory concentration (37 *µ*g·mL^−1^) on *P. aeruginosa* ATCC 9027. The lowest inhibitory activity was recorded by the butanolic fraction (1.67 mg·mL^−1^) on *S. aureus* ATCC 25923, followed by the ethyl acetate fraction (1.46 mg·mL^−1^) on *L. monocytogenes* ATCC 7644. The minimum bactericidal concentrations of the fractions ranged from 0.037 to 2.500 mg·mL^−1^ depending on the bacterial strain. Gentamicin showed the best MBC value on *Pseudomonas aeruginosa* ATCC 9027, followed by the butanolic fraction on the same strain. The ethyl acetate fraction gave the highest minimum bactericidal concentrations.

## 4. Discussion

The aim of this study was to investigate the antioxidant and microbiological potential of *U. chamae*, a food plant in the flora of Burkina Faso. Analyses showed that antioxidant activities varied according to fractions. The ethyl acetate and butanoic fractions showed the highest levels of phenolic compounds. Similarly, the butanoic fraction showed the best iron-reducing power and DPPH antiradical activity. This difference in content and activity between fractions can be explained by the polarity of the extracted compounds. Indeed, many authors have reported that plant components can be polar or nonpolar in nature. Phenolic compounds are more soluble in polar organic solvents due to the presence of a hydroxyl group. This may explain why the polar fractions (ethyl acetate and butanoic acid) showed the highest levels. Comparing the values obtained with those in the literature, the values obtained appear to be relatively higher than those reported by other authors. Indeed, the authors of [10] obtained total phenolic contents in the order of 110.51 *μ*g GAE/mg of extracts. The authors of [25] reported total flavonoid contents of 41.06 *μ*g ER/100 mg extract from aqueous extracts of *U. chamae* leaves. The difference in content between fractions makes it possible to identify the solvent that will optimize the extraction of bioactive compounds. The negative correlations recorded between the IC50 of DPPH activity and phenolic content could mean that free radical scavenging activity is linked to phenolic compounds. The presence of phenolic compounds in *U. chamae* leaves could be beneficial to health. In fact, phenolic compounds are capable of reducing the production of reactive oxygen species by neutrophils, thereby reducing the risk of disease. They can also inhibit enzymes involved in cancer cell activation. FRAP activity being negatively correlated with flavonoids and positively with phenolics could mean that the iron-reducing power of the extracts is linked to other types of phenolic compounds, notably phenolic acids or tannins other than flavonoids.

Significant antibacterial activity against the different strains and fractions of ethanolic extracts from *U. chamae* leaves was recorded. The diameters of the inhibition zones of our fractions were very close to those of [26] on *E. coli* ATCC 25922 and *S. aureus* ATCC 25923 who had recorded diameters ranging from 10 to 11 mm and 9 to 10 mm, respectively, with methanolic extracts (200 and 250 mg/mL), but at very higher concentrations than ours (5 and 10 mg/mL). The authors of [9] having worked on *U. manjensis*, from the same family as *U. chamae*, recorded inhibition diameters 7 mm smaller than ours on *E. coli* ATCC 25922, *B. cereus*, *S. aureus*, and *L. monocytogenes* and 6 mm on *P. aeruginosa* with decoction, aqueous macerate, and hot hydroalcoholic extract, respectively. They recorded diameters of 22 mm, 24 mm, and 10 mm more or less than our diameters on *S. Typhi* for hot aqueous extract, cold aqueous extract, and hot hydroalcoholic extract, respectively. The differences in strain sensitivity to extracts compared with other studies could be explained by origin, isolation techniques, strain characteristics, and handling techniques. Our results showed that all six bacterial strains were sensitive to gentamicin and butanolic and acetate ethyl fractions. Comparing the two fractions according to their respective concentrations, we find that the inhibition of the butanolic fraction is more or less superior to that of the ethyl acetate fraction. These results show that inhibition zones vary according to bacterial species and plant extract composition. Both fractions, at concentrations varying between 5 and 10 mg/mL, could have antibacterial potential on the strains tested. The interesting antibacterial properties of the plant studied could be explained by its phenolic and flavonoid compounds. Indeed, the ability of a herbal remedy to exert microbial growth inhibitory effects is due to chemical composition [27]. Phenolic compounds in general and flavonoids in particular are known for their diverse biological properties, notably antibacterial and antioxidant. The activity of these substances towards microorganisms can be exerted in a variety of ways. Some exert their activity by oxidizing or denaturing bacterial proteins [28]; others are more specific, altering membrane structures or inactivating key compounds or essential cell functions. These biomolecules are therefore bactericidal or bacteriostatic, depending on their nature and concentration [29]. *U. chamae* leaf extracts could therefore be used as dietary supplements to boost the immune system and prevent bacterial infections.

## 5. Conclusions

The study aimed to evaluate the antioxidant and antibacterial potentials of the fractions of the hydroethanolic extract of *Uvaria chamae* leaves, a food plant from Burkina Faso. The results showed that the butanol and ethyl acetate fractions showed the best antibacterial and antioxidant activity. In addition, significant and positive correlations were also recorded between phenolics and antioxidant activities by FRAP. The antibacterial and antioxidant activities of the active fractions would be related to their richness in bioactive compounds, including phenolics, which are powerful natural antioxidants. *U. chamae* leaf extracts could therefore be used as dietary supplements to boost the immune system and prevent bacterial infections.

## Figures and Tables

**Table 1:** Phenolics contents of different fractions.

Fractions	Phenolics (mg GAE/100 mg)	Flavonoids (mg QE/100 mg)
Hexanic	15.49 ± 0.18^b^	1.45 ± 0.12^d^
Dichloromethane (DCM)	17.79 ± 0.30^a^	1.89 ± 0.03^c^
Ethyl acetate (EAC)	18.33 ± 0.59^a^	5.93 ± 0.19^b^
Butanoic	18.13 ± 0.23^a^	7.26 ± 0.10^a^

The columns with different letters (a, b and c) are statistically different at the 5% level.

**Table 2:** IC50 of the different fractions.

Fractions or standard	IC50 (*µ*g·mL^−1^)
Hexanic	705 ± 2
Dichloromethane (DCM)	657 ± 4
Ethyl acetate (EAC)	379 ± 1
Butanoic	280 ± 5
Trolox	80 ± 5
Ascorbic acid	100 ± 3

**Table 3:** Pearson correlation between phenolics and antioxidant activities.

Variables	Total phenolics	Flavonoids	IC50 DPPH	FRAP
Total phenolics	**1**			
Flavonoids	0.717	**1**		
IC50 DPPH	−0.736	**−0.999**	**1**	
FRAP	0.161	−0.152	0.125	**1**

Values in bold are significant at the 5% level.

**Table 4:** Inhibition diameters of different fractions and gentamicin.

Bacterial strains	Inhibition diameters (mm)				Standard
	Ethyl acetate fraction		Butanoic fraction (mg·mL^−1^)		Gentamicin
	5 mg·mL^−1^	10 mg·mL^−1^	5 mg·mL^−1^	10 mg·mL^−1^	
*Bacillus cereus* ATCC 13061	12.00 ± 0.03^c^	11.17 ± 0.05^c^	17.00 ± 0.02^b^	17.17 ± 0.12^b^	32.01 ± 0.07^a^
*Listeria monocytogenes* ATCC 7644	12.08 ± 0.02^c^	14.12 ± 0.09^b^	15.15 ± 0.12^b^	16.67 ± 1.20^b^	36.09 ± 0.03^a^
*Staphylococcus aureus* ATCC 25923	10.50 ± 0.06^b^	11.50 ± 0.06^b^	11.17 ± 0.015^b^	13.50 ± 1.25^b^	31.01 ± 0.41^a^
*Escherichia coli* ATCC 8739	11.00 ± 0.01^b^	11.17 ± 0.21^b^	13.50 ± 1.27^b^	13.83 ± 1.25^b^	29.33 ± 0.12^a^
*Pseudomonas aeruginosa* ATCC 9027	12.33 ± 0.99^c^	11.17 ± 1.28^c^	16.00 ± 0.54^b^	12.50 ± 0.50^c^	27.00 ± 1.02^a^
*Salmonella typhi* ATCC 19430	12.33 ± 1.25^c^	12.50 ± 0.87^c^	14.09 ± 0.10^c^	18.33 ± 0.55^b^	34.00 ± 0.75^a^

The columns with different letters (a, b and c) are statistically different at the 5% level.

**Table 5:** Sensitivity of bacterial strains to fractions and gentamicin.

Bacterial strains	Inhibition diameters (mm)				Standard
	Ethyl acetate fraction		Butanoic fraction		Gentamicin
	5 mg·mL^−1^	10 mg·mL^−1^	5 mg·mL^−1^	10 mg·mL^−1^	
*Bacillus cereus* ATCC 13061	+	+	++	++	+++
*Listeria monocytogenes* ATCC 7644	+	+	++	++	+++
*Staphylococcus aureus* ATCC 25923	+	+	+	+	+++
*Escherichia coli* ATCC 8739	+	+	+	+	+++
*Pseudomonas aeruginosa* ATCC 9027	+	+	++	+	+++
*Salmonella typhi* ATCC 19430	+	+	+	++	+++

## Data Availability

The data utilized in this research have not been previously released or published in any form. The datasets employed and/or analysed during the present study can be obtained from the corresponding author.
